# A Missing Data Approach to Correct for Direct and Indirect Range Restrictions with a Dichotomous Criterion: A Simulation Study

**DOI:** 10.1371/journal.pone.0152330

**Published:** 2016-03-28

**Authors:** Andreas Pfaffel, Marlene Kollmayer, Barbara Schober, Christiane Spiel

**Affiliations:** Department of Applied Psychology: Work, Education, Economy, Faculty of Psychology, University of Vienna, Vienna, Austria; Hvidovre Hospital, DENMARK

## Abstract

A recurring methodological problem in the evaluation of the predictive validity of selection methods is that the values of the criterion variable are available for selected applicants only. This so-called range restriction problem causes biased population estimates. Correction methods for direct and indirect range restriction scenarios have widely studied for continuous criterion variables but not for dichotomous ones. The few existing approaches are inapplicable because they do not consider the unknown base rate of success. Hence, there is a lack of scientific research on suitable correction methods and the systematic analysis of their accuracies in the cases of a naturally or artificially dichotomous criterion. We aim to overcome this deficiency by viewing the range restriction problem as a missing data mechanism. We used multiple imputation by chained equations to generate complete criterion data before estimating the predictive validity and the base rate of success. Monte Carlo simulations were conducted to investigate the accuracy of the proposed correction in dependence of selection ratio, predictive validity, and base rate of success in an experimental design. In addition, we compared our proposed missing data approach with Thorndike’s well-known correction formulas that have only been used in the case of continuous criterion variables so far. The results show that the missing data approach is more accurate in estimating the predictive validity than Thorndike’s correction formulas. The accuracy of our proposed correction increases as the selection ratio and the correlation between predictor and criterion increase. Furthermore, the missing data approach provides a valid estimate of the unknown base rate of success. On the basis of our findings, we argue for the use of multiple imputation by chained equations in the evaluation of the predictive validity of selection methods when the criterion is dichotomous.

## Introduction

A recurring methodological problem in the evaluation of the predictive validity of selection methods is that the values of the criterion variable are available only for selected applicants. This loss of criterion data for non-selected applicants is an inherent effect of selection and is known as the *range restriction problem* [1–5]. The problem occurs, for example, in the evaluation of an admission test in higher education, because data on academic success are only available for applicants who are admitted to the program. As an effect of the selection, the sample of selected applicants is not random and therefore not representative of the applicant population. Consequently, the observed sample correlation is a biased estimate of the population correlation, i.e. of the predictive validity. The correlation between a predictor *X* and a criterion *Y* obtained from the (available) range restricted dataset (i.e., the selected sample) underestimates the correlation we would obtain from the (not available) unrestricted dataset. Hence, this biased sample correlation has to be corrected to provide a more valid population estimate.

Correction methods for the range restriction problem have been widely studied for continuous criterion variables [5–17]. However, sociological, medical, and psychological research often deal with dichotomous criterion variables [18,19]. Dichotomous variables are characterized by a division of the individuals of a sample or population into two groups. The division can be based on either a qualitative or a quantitative characteristic. In the former case, the dichotomous variable is labelled as natural, and in the latter case as artificial [20]. For example, in higher education, the graduation status of a student is naturally dichotomous (‘graduated’ versus ‘not graduated’). An artificially dichotomous variable is one that has a continuous underlying scale, but has been dichotomized (e.g., ‘high performers’ versus ‘low performers’). The few existing approaches [21,22] to correct the biased correlation in the case of a dichotomous criterion are inapplicable because they require information about the base rate of success, i.e. the proportion of successful individuals in the unrestricted dataset. However, this information is typically not available. Thus, there is a lack in scientific research on suitable correction methods and their accuracies when the criterion variable is dichotomous.

In the present paper, we aim to overcome this deficiency by viewing the range restriction problem as a missing data mechanism [14,23]. As there is comprehensive literature on dealing with missing data, we can draw on a variety of techniques and research results. This potential is a great advantage of this approach, which has not yet been used to correct for range restriction with a dichotomous criterion variable [24–26]. We apply this approach to the two most common selection scenarios in personnel selection and higher education [27]: the *direct range restriction* (DRR) scenario and the *indirect range restriction* (IRR) scenario. In a DRR scenario, selection is based directly on the predictor variable *X*, whereas in an IRR scenario, selection is based on another variable *Z*.

First of all, we describe the loss of data in the two selection scenarios DRR and IRR, show which data are used for the correction, and give a brief introduction to Thorndike’s well-known and widely used correction formulas in the case of a continuous criterion variable. Next, we provide a brief overview of methods for handling missing data and demonstrate that the proposed approach, *multiple imputation by chained equations*, is suitable for correcting for range restriction in both scenarios involving a dichotomous criterion. Then, we emphasize the critical role of the base rate of success, which has not been taken into account in previous approaches. Our proposed missing data approach generates complete data from which the base rate of success as well as the unbiased predictive validity can be obtained. Finally, we investigate the accuracy of the proposed correction by conducting Monte Carlo simulations, which allow for a comparison of the corrected parameters and the unbiased parameters (predictive validity and base rate of success) in an experimental design.

### Direct and indirect range restriction

The most straightforward selection scenario is the direct range restriction (DRR) scenario, or explicit selection, which is commonly referred to as Thorndike’s Case 2 [4,5]. In a DRR scenario, selection is based directly on the predictor variable *X* and occurs top down. The idea is that applicants with a higher value of *X* are more suitable, and thus more likely to have a higher value in *Y*. As selection is based on values of *X*, the range of *X* is restricted in the selected sample. For this reason, this methodological problem is called the range restriction problem. The variable *X* can be either a score in a single-selection method (e.g., a psychometric test), or a composite score derived from several selection methods (e.g., a psychometric test and a quantitative interview). For example, in higher education in Austria, prospective students of medicine are selected based solely on an entrance examination [28–30]. In the case of DRR, values of *X* are available for all applicants, whereas values of *Y* are only available for selected applicants.

The indirect range restriction scenario (IRR) occurs when applicants are selected on the basis of another variable *Z*, which is usually correlated with *X*, *Y*, or both. The IRR scenario or incidental selection is commonly referred to as Thorndike’s Case 3 [4,5]. Although selection is based on *Z*, the predictive validity of *X* remains of interest. Suppose a selection procedure consists of a psychometric test and a quantitative interview, and we want to assess the predictive validity of the psychometric test, the predictor *X*. For selection, we use the composite score *Z* derived from both selection methods. Organizations often use a composite score for selection and need to know the predictive validity of a single selection method in order to increase the predictive validity of the whole selection procedure (e.g., by removing or weighting a particular selection method). In the case of IRR, values of *X* and *Z* are available for all applicants, whereas values of *Y* are available for selected applicants only.

**Fig 1:**
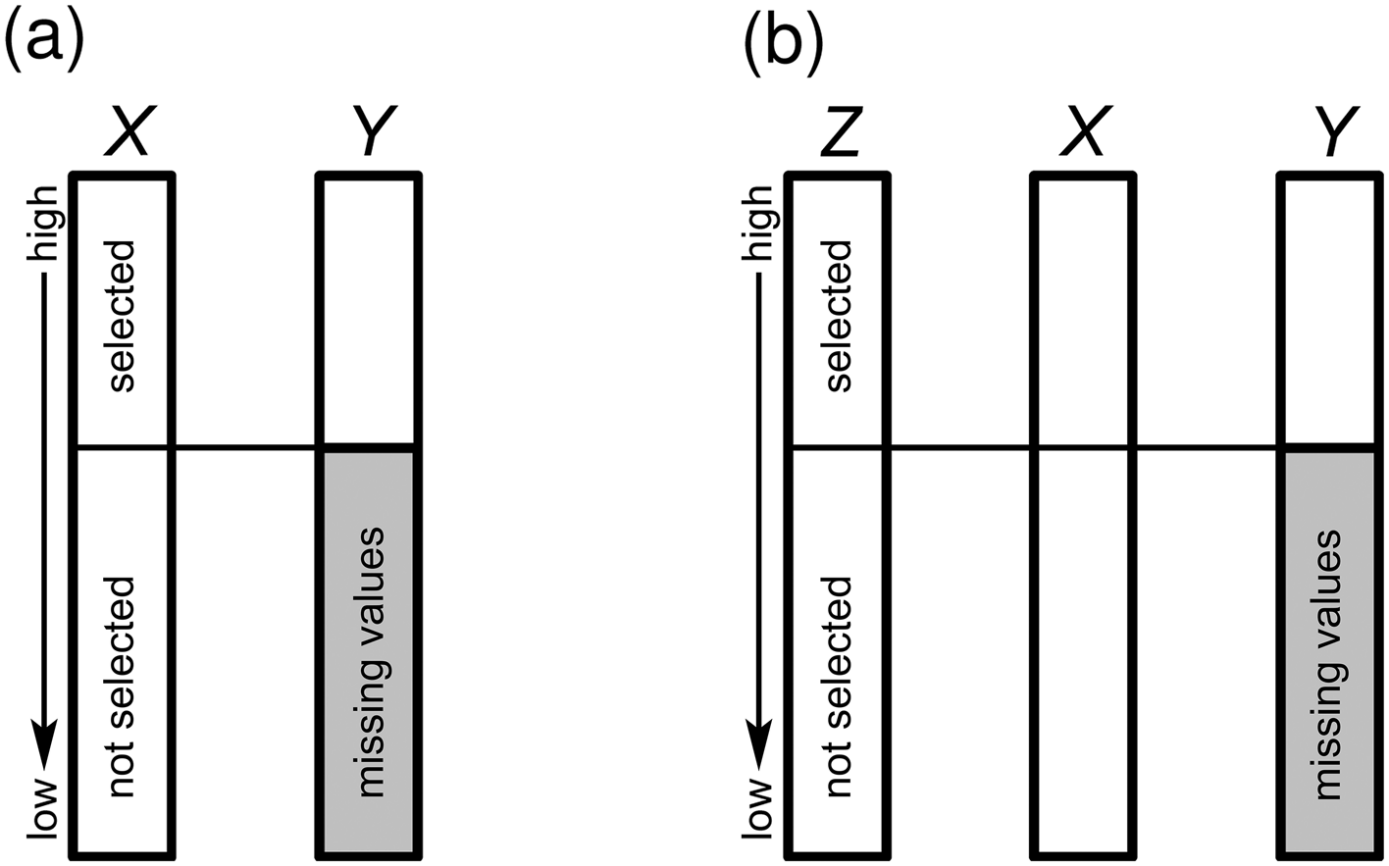
Missing data patterns under range restriction. **(a)** Direct range restriction scenario (selection on *X*), and **(b)** indirect range restriction scenario (selection on *Z*). The shaded areas in *Y* represent the location of the missing values in the dataset.

The amount of data loss depends on the selection ratio (SR), which is defined as the ratio of available places to the number of applicants. The SR ranges between 0 and 1, or between 0% and 100%. For example, if 200 study places are available and 500 applicants apply for them, the SR is 200 divided by 500 or 40%. The top 40% of applicants will be selected and 60% will be unselected. Hence, in this case we have missing values in the criterion variable *Y* for 60% of the applicants, but no missing values in *X* or *Z*. Fig 1 shows the missing data pattern for a SR of 40% in the cases of DRR and IRR.

In both scenarios, the observed sample correlation between *X* and *Y* is smaller than the correlation we would obtain from the unrestricted dataset, i.e. the predictive validity of the selection method is underestimated. To overcome this problem in the case of a continuous criterion variable, Thorndike [5] presented formulas to correct the Pearson correlation coefficient for DRR and IRR. The goal of these correction formulas is to estimate the correlation in the unrestricted dataset, which is the best estimate available of the population correlation, based on the correlation obtained from the restricted dataset. Correction formulas are commonly applied in predictive validity studies of large-scale testing programs, such as the Graduate Record Examination (GRE) [31,32], the Scholastic Aptitude Test (SAT) [33,34], and the Graduate Management Admission Test (GMAT) [35]. Correction formulas are also applied in other fields, e.g. in predicting job performance [36], and in evaluating the selection of pilot candidates in the US Air Force [37].

The formula for correcting for direct range restriction (DRR) presented by Thorndike is:

$$\rho_{XY}=\frac{\left({\text{S}}_{X}/{\text{s}}_{X}\right)r_{XY}}{\sqrt{1+r_{XY}^{2}\left({\text{S}}_{X}^{2}/{\text{s}}_{X}^{2}-1\right)}},$$ (1)

where ρ_*XY*_ is the true or unrestricted correlation coefficient, *r*_*XY*_ is the biased Pearson correlation coefficient obtained from the restricted dataset, and *s*_*X*_ and *S*_*X*_ are the standard deviations of *X* for the restricted and the unrestricted datasets [4]. The formula for correcting for indirect range restriction (IRR) is:

$$\rho_{XY}=\frac{r_{XY}+r_{ZX}\cdot r_{ZY}\left(S_{Z}^{2}/s_{Z}^{2}-1\right)}{\sqrt{1+r_{ZX}^{2}\left(S_{Z}^{2}/s_{Z}^{2}-1\right)}\;\cdot \sqrt{1+r_{ZY}^{2}\left(S_{Z}^{2}/s_{Z}^{2}-1\right)}},$$ (2)

where *r*_*XY*_, *r*_*ZX*_, and *r*_*ZY*_ are the uncorrected Pearson correlation coefficients obtained from the restricted dataset, and *s*_*Z*_ and *S*_*Z*_ are the standard deviations of variable *Z* for the restricted and the unrestricted dataset [4]. The core term in both correction formulas is the ratio of the standard deviations of the selection variable (*X* or *Z*).

The two formulas require that the assumption of linearity between *X* and *Y* as well as the assumption of homoscedasticity hold. In psychometric literature, it is well documented that corrected correlations are less biased than uncorrected correlations, even over a wide range of assumption violations [9,17,38,39]. Correcting for range restriction is recognized as professional practice because the corrected correlation coefficient is generally the best estimate of the population validity coefficient [40]. In general, the accuracy of the correction increases as the selection ratio increases and as the predictive validity increases [41]. Whereas Thorndike’s correction formulas have been widely studied for continuous criterion variables, they have not been studied for dichotomous ones. Therefore, we investigate how usable Thorndike’s correction formulas are in the case of a dichotomous criterion variable. Furthermore, we propose an approach based on state of the art methods for dealing with missing values that has not yet been applied in predictive validity studies [23].

### Range restriction as a missing data mechanism

First, we give a brief overview of missing data mechanisms to locate the range restriction problem in this line of research. Afterwards, we introduce different methods of handling missing data and propose an approach for handling missing values in dichotomous dependent variables. One advantage of viewing the range restriction as a missing data mechanism is that we can draw on a variety of techniques and research results in dealing with missing data [24,25,42]. However, this approach is seldom used with range restriction problems [14].

Rubin [43] describes three mechanisms essential as assumptions in dealing with missing values. These three mechanisms describe how the probability of a missing value relates to the data [24]: (1) Missing completely at random (MCAR) means the probability of missing data on *Y* is unrelated to other measured variables and is unrelated to the values of *Y* itself; (2) Missing at random (MAR) means the probability of missing data on *Y* is related to some other measured variable (or variables) in the analysis model but not to the values of *Y* itself; and (3) Missing not at random (MNAR) means the probability of missing data on *Y* is related to the values of *Y* itself, even after controlling for other variables. We consider both selection scenarios to be MAR, because the probability of missing values on *Y* depends either on *X* in the case of DRR, or on *Z* in the case of IRR, and not on values of *Y* itself. In other words, there is no relationship between the probability of missing values on *Y* and the values of *Y* after partialling out other variables. In the case of a MAR mechanism, we can estimate the missing values based on the observed values [24].

Over the past few decades, methodologists have proposed different techniques for dealing with missing data. Many of these approaches have enjoyed widespread use, but several of them, like listwise or pairwise deletion and single imputation techniques (e.g., arithmetic mean imputation, single regression imputation, and single EM imputation) are no longer considered to be state of the art, because they have potentially serious drawbacks [24]. Listwise and pairwise deletion require an MCAR mechanism, and produce biased parameter estimates with MAR and MNAR data. Deletion of incomplete cases can reduce the statistical power dramatically, even when the data are MCAR. Single imputation techniques also produce biased parameter estimates with MAR data and attenuate standard errors. Single regression imputation and single EM imputation overestimate correlations and attenuate variances and covariances, even when the data are MCAR, because they impute the data with perfectly correlated scores [24,44]. In a single regression imputation, all imputed values fall directly on the regression line and therefore lack variability. In contrast, arithmetic mean imputation attenuates correlations. Consequently, single imputation techniques are not suitable for many reasons, especially with regard to estimating correlation coefficients.

The two approaches that methodologists currently regard as state of the art [25,26] are (1) full information maximum likelihood (FIML), and (2) multiple imputation (MI). Neither of these approaches suffers from the problems mentioned for deletion of incomplete cases and single imputation techniques. The FIML approach estimates the most plausible parameters of a statistical model given the data. In other words, the goal is to identify the population parameter values with the highest probability of producing the data of a certain sample. The population parameter values are estimated with iterative optimization algorithms (e.g., expectation maximization algorithm). For a detailed description of likelihood-based approaches, see Little and Rubin [25], or for a less technical description see Enders [24].

The second state of the art approach to handle missing data problems is multiple imputation (MI) [24,25,45]. A multiple imputation analysis consists of three distinct steps: the imputation phase, the analysis phase, and the pooling phase. The imputation phase creates several complete datasets (e.g., *m* = 20 imputations) based on one dataset with missing values. Each of these complete datasets contains different plausible estimates of the missing values, but identical values for the observed data. In the analysis phase, data can be analyzed with conventional statistical methods, but the analysis has to be performed *m* times, once for each complete dataset. The goal of the pooling phase is to combine the *m* parameter estimates into a single set of parameter estimates. The pooled parameter estimate is simply the arithmetic average of the *m* estimates from the analysis phase [46]. Analyzing multiple datasets and pooling the results sounds laborious, but modern MI software packages automate this procedure.

FIML und MI make the same assumptions (MAR and multivariate normality), have similar statistical properties, and frequently produce equivalent results [24,42]. Despite making the same assumptions, the two approaches differ in their mathematical background: The mathematical background of FIML is maximum likelihood estimation, whereas MI is based on Bayesian estimation. Therefore, there are important differences between the two approaches. While FIML maximizes the likelihood function to estimate the parameters without replacing missing values, MI replaces the missing values before estimating the parameters from the complete datasets. In contrast to FIML, MI effectively separates the imputation and the analysis phase. This may yield to different parameter estimates between the two approaches. Typically, in MI the imputation model includes many variables of the dataset, whereas the analysis model includes a subset of these variables.

Real data often do not conform to the modeling assumption of multivariate normality. Real data might be skewed, not negative, or bimodal, to name just a few deviations from normality. This mismatch between the distribution of the observed and imputed data may adversely affect the estimates of interest. MI generally tends to be robust against violations of normality [45,47,48]. Deviations from the normal distribution have a small effect on estimates that rely on the center of the distribution, like mean or regression coefficients, but may have significant effects on variances. Demirtas et al. [48] found that MI performs accurately with regard to the mean structure of skewed or multimodal distributions in large samples (n = 400), even for 75% missing values.

In the present study, we have to handle missing values in a dichotomous dependent variable. In light of this, we want to give a conceptual overview of Bayesian multiple imputation using logistic regression, which is considered to handle dichotomous variables most efficiently. Imputation of incomplete dichotomous variables is possible under the broad class of generalized linear models (GLM) [49]. The logistic regression models the probability that *Y*_*i*_ = 1 given *X*_*i*_ and model parameter vector *β* as [45]:

$$\text{Pr}\left(Y_{i}=1|X_{i},\beta \right)=\frac{\text{exp}\left(X_{i}\beta \right)}{1+\text{exp}\left(X_{i}\beta \right)}$$ (3)

The general idea is to estimate the probability model on the subset of the observed data (i.e., the restricted sample), and to impute the missing values with plausible values according to the fitted probabilities. For example, a probability of .80 means that *Y*_*i*_ has a chance of 80% of becoming 1 and a 20% chance of becoming 0. For a large number of imputations, the percentage of datasets in which *Y*_*i*_ = 1 tends towards 80%. The Bayesian method draws *β* from its respective posterior distributions. The posterior distribution contains the variability of *β* that needs to be incorporated into the imputations. In Bayesian statistics, Markov chain Monte Carlo (MCMC) methods are used to find the posterior distribution of the parameters. MCMC algorithms draw samples from probability distributions based on constructing a Markov chain that has the desired distribution as its stationary distribution. The state of the chain after a very large number of steps is then used as a sample of the desired distribution. The quality of this sample increases with the number of steps. For a mathematical description of the Bayesian logistic regression imputation model, see Rubin [46].

Currently, MI is generally accepted as the best method for dealing with incomplete data in many fields [45]. Therefore, we suggest using multiple imputation by chained equations (MICE) to correct for range restriction in cases of DRR and IRR when the criterion variable is dichotomous. The proposed missing data approach first replaces the missing values of the criterion variable *Y* and generates several complete (unrestricted) datasets. Then, the correlation coefficient can be calculated based on these complete datasets.

### The critical role of the base rate of success

A very important factor to be considered when correcting for range restriction with a dichotomous criterion is the base rate of success (BR) [21]. The BR is the percentage of individuals who would be successful on the criterion if there were no selection. The BR is calculated by dividing the number of successful individuals by the number of applicants, and ranges between 0 and 1, or between 0% and 100%. The BR contains unbiased information about the proportions of cases in the categories *p* (*Y* = 0; not successful) and *q* (*Y* = 1; successful) of a dichotomous criterion variable. For example, if all applicants are admitted to a study program and 60% of them complete this study program successfully, the BR is .6, or 60%.

Unfortunately, in the case of selection, the BR is unknown, as we cannot obtain the percentage of unselected applicants able to succeed on the criterion. We can only obtain the *success rate* of the selected sample, which is the number of successful individuals divided by the number of selected applicants. The success rate, however, is a biased estimator for the BR. Assuming a selection method determines the most suitable applicants, we will obtain a success rate which is higher than the BR.

Next, we will show how the two proportions *p* and *q* constituting the BR affect the magnitude of the correlation coefficient between a continuous variable *X* and a dichotomous variable *Y* [50,51]. Two correlation coefficients can be distinguished depending on whether the dichotomous variable is based on a qualitative or on a quantitative characteristic. In the former case, the dichotomous variable is labelled as natural, and in the latter case as artificial [20]. For a naturally dichotomous variable, the point-biserial correlation coefficient ρ_pb_ is used [50,51]:

$$\rho_{\text{pb}}=\frac{\left(M_{1}-M_{0}\right)\sqrt{pq}}{\sigma_{X}},$$ (4)

where *M*_1_ and *M*_0_ are the mean values of the continuous variable *X* for the group ‘not successful’ (*Y* = 0) and the group ‘successful’ (*Y* = 1), and σ_*X*_ is the standard deviation of *X*. *p* and *q* = 1 − *p* represent the proportions of the two groups ‘not successful’ and ‘successful’. ρ_pb_ ranges between -1 and +1. Normality of *X* is an assumption for significance testing, but not for calculating ρ_pb_.

An artificially dichotomous variable is created whenever the values of a continuous variable are divided into two groups at a specific cut-off point. For example, student performance is measured on a continuous scale, and students are divided into ‘low’ and ‘high’ performers on the basis of their performance. In this case, a biserial correlation coefficient ρ_b_ is the more appropriate calculation. In the case of an artificially dichotomous variable, ρ_pb_ systematically underestimates the Pearson correlation coefficient which would have been obtained before dichotomization [41]. ρ_b_ is related to ρ_pb_ as shown in Formula 5:

$$\rho_{\text{b}}=\rho_{\text{pb}}\frac{\sqrt{pq}}{h},$$ (5)

where *h* is the ordinate of the standard normal distribution at the point at which the cut for the dichotomization was made.

Both ρ_pb_ and ρ_b_ depend on the proportions *p* and *q*. As *p* and *q* become more extreme (e.g., .1 and .9), the correlation coefficient becomes smaller. Different values of the BR and the success rate result in different values of the correlation coefficients. Hence, *p* and *q* as obtained from the restricted dataset are different from the *p* and *q* we would obtain from the unrestricted dataset. Therefore, using the success rate to estimate the predictive validity results in biased correlation coefficients.

Two approaches have been proposed so far to assess the predictive validity of a selection method when the criterion variable is dichotomous. However, both approaches assume that the BR is known (e.g., from the literature), or should be assumed. The first approach is to apply the Taylor-Russell tables for a dichotomous criterion variable [21]. These tables indicate values of ρ_pb_ for the combination of the SR, the success rate, and the BR. The value for ρ_pb_ can only be taken from the tables if the values for the other three parameters are known. While the SR and the success rate are typically known, the BR is not and must be assumed. The second approach focuses on the effect size Cohen’s *d* as a measure of the predictive validity in the case of a naturally dichotomous variable, and offers correction formulas for DRR and IRR [22]. The formulas correct the biased effect size *d* (obtained from the selected sample) into an unbiased *d* using the ratio of the unrestricted standard deviation to the restricted standard deviation, as known from Thorndike’s correction formulas. Both formulas to calculate the unbiased *d* require the BR, which must be known or assumed. However, assuming the BR is an arbitrary approach, and different assumptions of the BR result in different values of the predictive validity, i.e. different values of the correlation coefficients.

So far, the scientific literature does not provide any correction method for situations in which the BR is unknown. However, when correcting for range restriction with a dichotomous criterion, both the biased success rate and the biased correlation have to be considered. The proposed missing data approach allows for this, as it generates complete datasets from which the BR as well as the unrestricted correlation can be obtained. Therefore, the proposed approach provides an empirical estimation for both the correlation coefficients and the BR based on the selected sample.

### Purposes of this Study

Correction methods for range restriction have been studied almost exclusively for continuous criterion variables. Therefore, the aim of the present study was to give empirical evidence in an experimental design on correcting for direct range restriction (DRR) and indirect range restriction (IRR) when the criterion variable is dichotomous.

The first purpose is to compare the two approaches (1) multiple imputation by chained equations (MICE), and (2) Thorndike’s correction formulas (Formulas 1 & 2) with regard to the accuracy of the correction of the biased sample correlations.

The second purpose is to investigate the effect of a weak, moderate, and strong relationship between predictor and criterion on the accuracy of the correction of the biased sample correlations with multiple imputation by chained equations. Studies investigating Thorndike’s correction formulas have shown that the accuracy of the correction increases as the correlation between predictor and criterion increases.

The third purpose is to investigate the accuracy of the correction of the biased BR with multiple imputation by chained equations. Previous approaches are less useful when the criterion variable is dichotomous because they do not consider that the success rate is a biased estimate for the unknown BR. However, the proposed missing data approach allows us to estimate the BR.

The fourth purpose of this study is to investigate the effect of the strength of the correlation between *Z* and *X* on the accuracy of the correction with multiple imputation by chained equations in an IRR scenario.

## Method

### Procedure

We conducted Monte Carlo simulations to investigate the two correction approaches: a) Thorndike’s correction formulas, and b) multiple imputation by chained equations (MICE) in an experimental design using the program R Statistics [52]. We wrote four R scripts (see S1–S4 Rscripts) in order to conduct the Monte Carlo simulations for the following four conditions: 1. DRR with an artificially dichotomous criterion variable; 2. DRR with a naturally dichotomous criterion variable; 3. IRR with an artificially dichotomous criterion variable; and 4. IRR with a naturally dichotomous criterion variable. The Monte Carlo simulations were conducted with 5,000 iterations for each condition. The procedure for the Monte Carlo simulation consisted of the following steps.

#### Step 1—Data simulation

We generated 5,000 unrestricted multivariate datasets (sample size *N* = 500) for each condition by varying the correlation coefficient between *X* and *Y* from .10 to .90 and the base rate of success (BR) from 10% to 90%. In the case of IRR, there was a third variable *Z*, meaning that we varied not only the correlation coefficient between *X* and *Y* but also the correlations between *Z* and *X*, and Z and *Y* from .10 to .90.

#### Step 2—Selection

We simulated the selection for nine levels of the selection ratio (SR) ranging from 10% to 90% with step width 10% (which corresponded to missing values in *Y* from 90% to 10%). This yielded 5000 * 9 = 45000 restricted datasets. In the case of DRR, datasets were sorted in descending order by *X*; in the case of IRR, in descending order by *Z*. We selected those cases with the highest values in *X* (DRR), and with the highest values in *Z* (IRR). The percentage of selected cases depended on the SR. Values of *Y* for non-selected cases were deleted (i.e., converted into missing values). The range restricted or selected samples created in this way were saved into new datasets and were used for applying the correction.

#### Step 3—Correction

Both approaches were applied to the range restricted datasets. In the first approach, Thorndike’s correction formulas for DRR (Eq 1) and IRR (Eq 2) were used to calculate the estimate of the correlation coefficient between predictor *X* and criterion *Y*. In the second approach, we used multiple imputation by chained equations to generate *m* = 20 imputed datasets (see subsection Imputation of the missing values). For each imputed dataset, we calculated the correlation coefficient between *X* and *Y*, and the BR. The MI analysis pools the *m* = 20 estimates into a single point estimate. Rubin [46] showed that the multiple imputation point estimate is the arithmetic mean of the *m* estimates.

#### Step 4—Analysis of parameter estimates

In order to analyse the accuracy of the correction, we compared the parameter estimates of both approaches with the true parameters obtained from the unrestricted dataset. All estimates of the parameters are denoted with the accent symbol hat, where ${\hat{r}}_{\text{b}}$ is the estimate of the biserial correlation coefficient and ${\hat{r}}_{\text{pb}}$ is the estimate of the point-biserial correlation coefficient. We calculated the residual of each parameter estimate. For example, the residual for the point-biserial correlation coefficient was ${\hat{r}}_{\text{pb}}-\rho_{\text{pb}}$, where ρ_pb_ was the true unrestricted correlation coefficient.

When running Monte Carlo simulations, extreme conditions typically cause problems in statistical analysis. Consequently, marginal conditions have to be defined. The logistic regression could not be applied when *Y* was constant. This was particularly likely to be the case when the BR and the correlation between *X* and *Y* were high and the SR was small. Therefore, a minimum variance in *Y*, or a minimum number of observations with *Y* = 1 (or *Y* = 0) was a necessary precondition for a valid estimate. We determined that a minimum of five observations in the two categories (‘successful’ and ‘not successful’) was a sufficient number of observations in the restricted dataset. This minimum number of observations was based on the rule of thumb in chi-square statistics for contingency tables. Therefore, we excluded samples that did not meet this prerequisite.

### Data simulation

We simulated multivariate data for two kinds of dichotomous criterion variables: a) an artificially dichotomous variable, and b) a naturally dichotomous variable. Both kinds of dichotomous criterion variables were simulated for a DRR and an IRR scenario. Purpose 1 was to compare the accuracy of the two approaches (Thorndike and MICE) for all possible combinations influencing the accuracy (ρ_*XY*_, ρ_ZX_, ρ_*ZY*_, BR, SR). Therefore, we generated the data using uniform random values for the correlation coefficients, and for the BR, both varied continuously from .1 to .9. The continuous variation of the factors facilitated the subsequent calculation of estimates aggregated over all factors and factor levels. On the basis of these aggregated estimates, the comparison of the two approaches can be displayed more clearly than based a large number of factor combinations.

a) We generated a bivariate standard normal distribution (DRR) or a trivariate standard normal distribution (IRR) using the mvrnorm() function of the MASS package [53]. Table 1 shows the design of the intercorrelation matrix for the DRR and IRR scenarios. In order to create an artificially dichotomous criterion variable *Y*, we dichotomized one of the standard normally distributed variables at a specific cut-off point. The cut-off point corresponded to the BR, which represented the number of ‘successful’ and ‘not successful’ individuals. Values higher than the cut-off point were coded as 1 (‘successful’); all other values were coded as 0 (‘not successful’). For example, a cut-off point at zero (= mean of the standard normal distribution) represented a BR of 50% (*p* = *q* = .50).

**Table 1:** Design of the intercorrelation matrix of the correlation coefficients for direct range restriction (DRR) and indirect range restriction (IRR).

DRR			IRR			
	*X*	*Y*		*X*	*Y*	*Z*
*X*	1	*r*_b_/*r*_pb_	*X*	1	*r*_b_/*r*_pb_	*r*
*Y*	*r*_b_/*r*_pb_	1	*Y*	*r*_b_/*r*_pb_	1	*r*_b_/*r*_pb_
			*Z*	*r*	*r*_b_/*r*_pb_	1

*X* is the predictor variable; *Y* is the dichotomous criterion variable; *Z* is the selection variable in the case of IRR; *r*_b_ is the biserial correlation coefficient; *r*_pb_ is the point-biserial correlation coefficient; *r* is the Pearson correlation coefficient.

b) In the case of a naturally dichotomous criterion, we simulated multivariate data based on a dichotomous variable (*Y*) and (for *X* and *Z*) a mixture of two univariate normal distributions, one normal distribution for each of the two criterion groups. This kind of data was used to develop the Taylor-Russell tables for a dichotomous criterion variable [21]. We followed this approach to be consistent with the literature on evaluating the predictive validity of a selection method when the criterion is dichotomous. First, we generated *Y* with the proportions of ‘successful’ and ‘not successful’ individuals based on the BR. Second, we generated two normally distributed variables (*X*_0_ and *X*_1_; one for each criterion group) with standard deviations of 1, and a mean difference M_1_ − M_0_. The mixture of *X*_0_ and *X*_1_ was the distribution of the continuous variable *X*. The mean difference is related to the amount of the point-biserial correlation coefficient ρ_pb_. The higher the mean difference, the higher ρ_pb_ (for constant BR, and constant standard deviations of *X*_0_ and *X*_1_). For example, when *X*_0_ and *X*_1_ are normally distributed with standard deviations of 1, the mean difference is 1.5, and the BR is 50%. In this example, the standard deviation of *X* is 1.25, resulting in a point-biserial correlation coefficient $\rho_{\text{pb}}=1.5\cdot \sqrt{.25}/1.25=.60$ (see Eq 4). For details on how to calculate σ_*X*_ for a mixture of two normal distributions in an analytical way, see Cohen [54]. With this procedure, the data simulation was completed for the DRR scenario. For the IRR scenario, we added the third variable *Z* in the same way as *X*, where *Z* was correlated with *X* and with *Y*. Li and colleagues also used this three-variable design in their Monte Carlo simulations to estimate the bootstrapped standard error of the Pearson correlation coefficient of Thorndike’s correction formula for IRR [7].

### Imputation of the missing values

We used the R package *MICE* (multivariate imputation by chained equations; version 2.22 [55]) to implement multiple imputation using fully conditional specification. The MICE package supports multivariate imputations of continuous data, binary data, unordered categorical data, and ordered categorical data. The algorithm imputed an incomplete variable by generating plausible values given other variables in the dataset. For the imputation of the dichotomous criterion variable, we used a Bayesian logistic regression implemented using the elementary imputation method logreg() of the MICE package. The imputation method logreg() was used with default specifications for the prior distributions and the Markov Chain Monte Carlo simulation (MCMC). Conventional wisdom suggests that multiple imputation analysis requires about *m* = 5 imputations [46,47]. This number of imputations was derived solely by considering the relative efficiency [24,46]. Contrary to this conventional wisdom, simulation studies show that only analyses based on *m* = 20 imputations yield comparable power to a maximum likelihood analysis and are therefore sufficient for many situations [24,42].

In our simulation study, we investigated samples with a rate of missing values up to 90% (corresponding to a SR of 10%). Therefore, we conducted a preliminary study to investigate the impact of the number of imputations on the accuracy of the parameter estimation dependent on the rate of missing values. We conducted Monte Carlo simulations using *m* = 5, 20, and 50 imputations for samples with rates of missing values of 70%, 50%, and 30% (*N* = 500). Table 2 shows the results of the preliminary study that *m* = 20 imputations provided a more accurate estimate than only *m* = 5 imputations. However, increasing the number of imputations beyond 20 provided no relevant improvement in the accuracy of the estimates. Therefore, we used *m* = 20 imputations in each simulation.

**Table 2:** Results of the preliminary study: Root mean square errors of the correlation estimates using *m* = 5, 20, and 50 imputations for 70%, 50%, and 30% missing values (DRR scenario, *N* = 500, 1000 iterations).

	${\hat{r}}_{\text{b}}$			${\hat{r}}_{\text{pb}}$		
*m*	70%	50%	30%	70%	50%	30%
5	.163	.118	.075	.112	.082	.049
20	.161 (-.002)	.106 (-.012)	.067 (-.008)	.105 (-.007)	.073 (-.009)	.044 (-.005)
50	.158 (-.003)	.104 (-.002)	.070 (.003)	.106 (.001)	.070 (-.003)	.042 (-.002)

${\hat{r}}_{\text{b}}$ is the estimate of the biserial correlation coefficient, ${\hat{r}}_{\text{pb}}$ is the estimate of the point-biserial correlation coefficient, *m* is the number of imputations. Values in brackets show the change in the RMSE as a result of the additional imputations.

### Analysis of parameter estimates

For our purpose of investigating the accuracy of the correction methods, we calculated the residual of each parameter estimate (Step 4 of the procedure). Accuracy is defined as the closeness of the estimated value to the true value of the parameter being estimated [56]. If the residual of a parameter estimate is close to zero, a correction method provides a very good estimation of the true parameter obtained from the unrestricted dataset. The concept of accuracy encompasses both precision (random error) and trueness (bias or systematic error), and therefore provides important quantitative information about the goodness of the correction. We used the root mean square error (RMSE) as a measure of precision, and the mean error (ME) as a measure of trueness. Let $\hat{\theta }$ be the parameter estimate and *θ* the true parameter, then the

$$RMSE=\sqrt{\frac{1}{n}\sum_{i=1}^{n}{\left({\hat{\theta }}_{i}-\theta \right)}^{2}},$$ (6)

and the

$$ME=\frac{1}{n}\sum_{i=1}^{n}\left({\hat{\theta }}_{i}-\theta \right).$$ (7)

The RMSE provides information about the probability that a correction is close to the true value. A small RMSE represents a small random error, i.e. a correction with high precision. The ME is the sample arithmetic mean of the residuals. An estimate is biased if the ME is different from zero. A positive ME represents an overestimation, and a negative ME represents an underestimation of the true parameter value.

We used *F*-ratio tests to compare Thorndike’s correction formulas with our proposed missing data approach in terms of the precision of the two estimates ${\hat{r}}_{\text{pb}}$ and ${\hat{r}}_{\text{b}}$. The *F*-ratio compares the mean square errors (MSEs), i.e. the variances of the residuals of the two approaches. For example, an *F*-ratio of 1 means that both correction methods have equal precision, while an *F*-ratio of 2 means that one correction method is twice as precise as the other one.

In order to investigate the effect of the strength of the relationship between predictor *X* and criterion *Y* (Purpose 2) and between selection variable *Z* and predictor *X* (Purpose 4) on the accuracy of the correction with multiple imputation by chained equations, we partitioned the true correlation coefficients obtained from the unrestricted dataset into three levels: a weak relationship (from .10 to < .40), a moderate relationship (from .40 to < .70), and a strong relationship (from .70 to .90). We compared the RMSEs of these three levels in order to demonstrate how the strength of the relationship between predictor and criterion affected the precision of the estimation.

## Results

**Fig 2:**
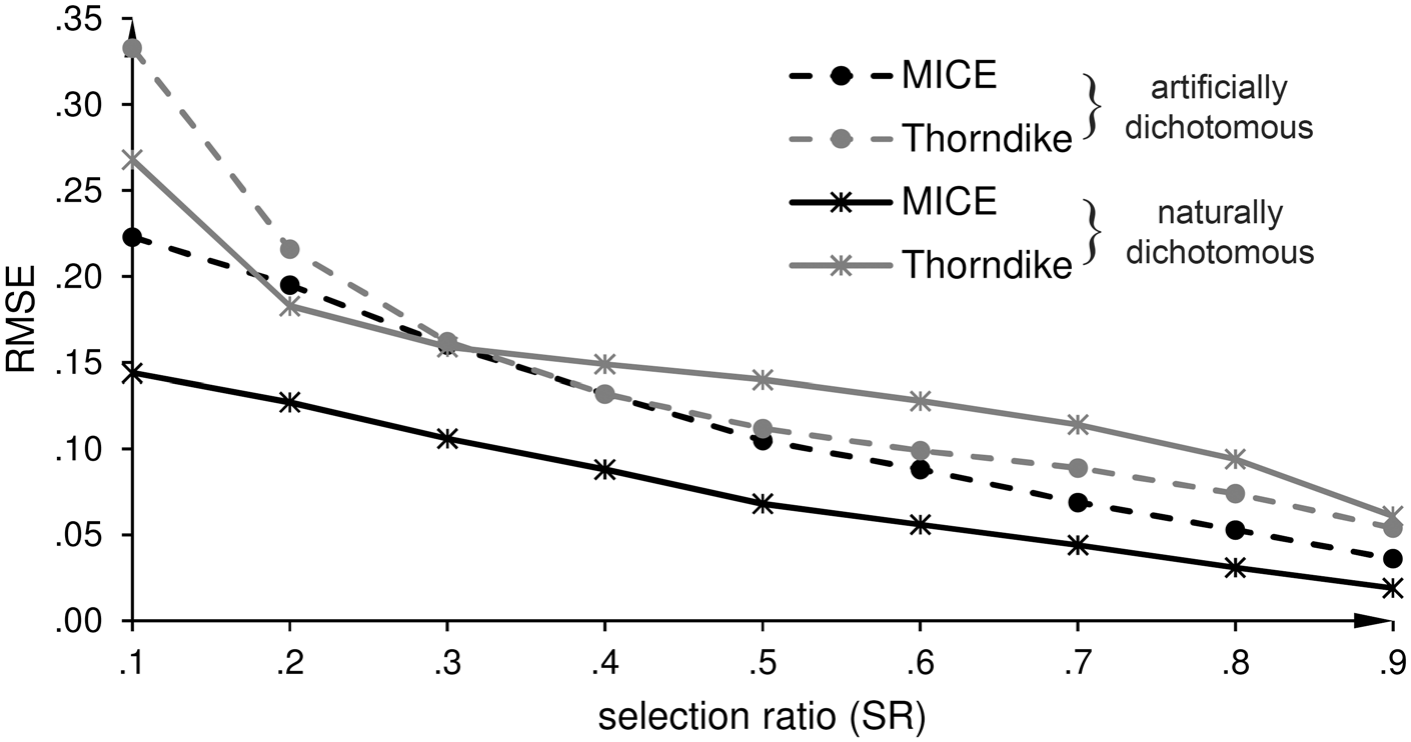
Direct range restriction (DRR): Root mean square error (RMSE) of the estimates of the predictive validity (${\hat{r}}_{\text{b}}$ and ${\hat{r}}_{\text{pb}}$). **${\hat{r}}_{\text{b}}$** is the estimate of the biserial correlation coefficient for an artificially dichotomous criterion variable, and ${\hat{r}}_{\text{pb}}$ is the estimate of the point-biserial correlation coefficient for a naturally dichotomous criterion variable.

**Fig 3:**
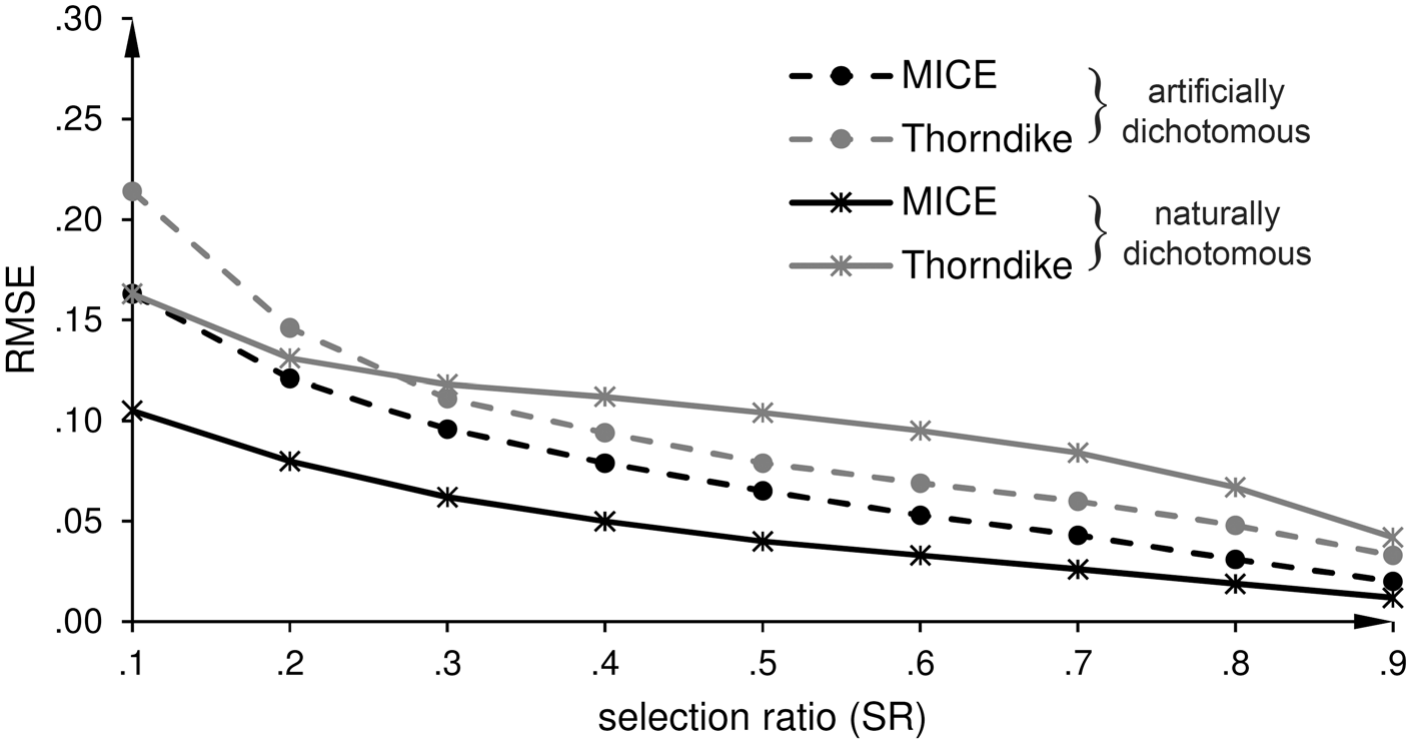
Indirect range restriction (IRR): Root mean square error (RMSE) of the estimates of the predictive validity (${\hat{r}}_{\text{b}}$ and ${\hat{r}}_{\text{pb}}$). ${\hat{r}}_{\text{b}}$ is the estimate of the biserial correlation coefficient for an artificially dichotomous criterion variable, and ${\hat{r}}_{\text{pb}}$ is the estimate of the point-biserial correlation coefficient for a naturally dichotomous criterion variable.

**Fig 4:**
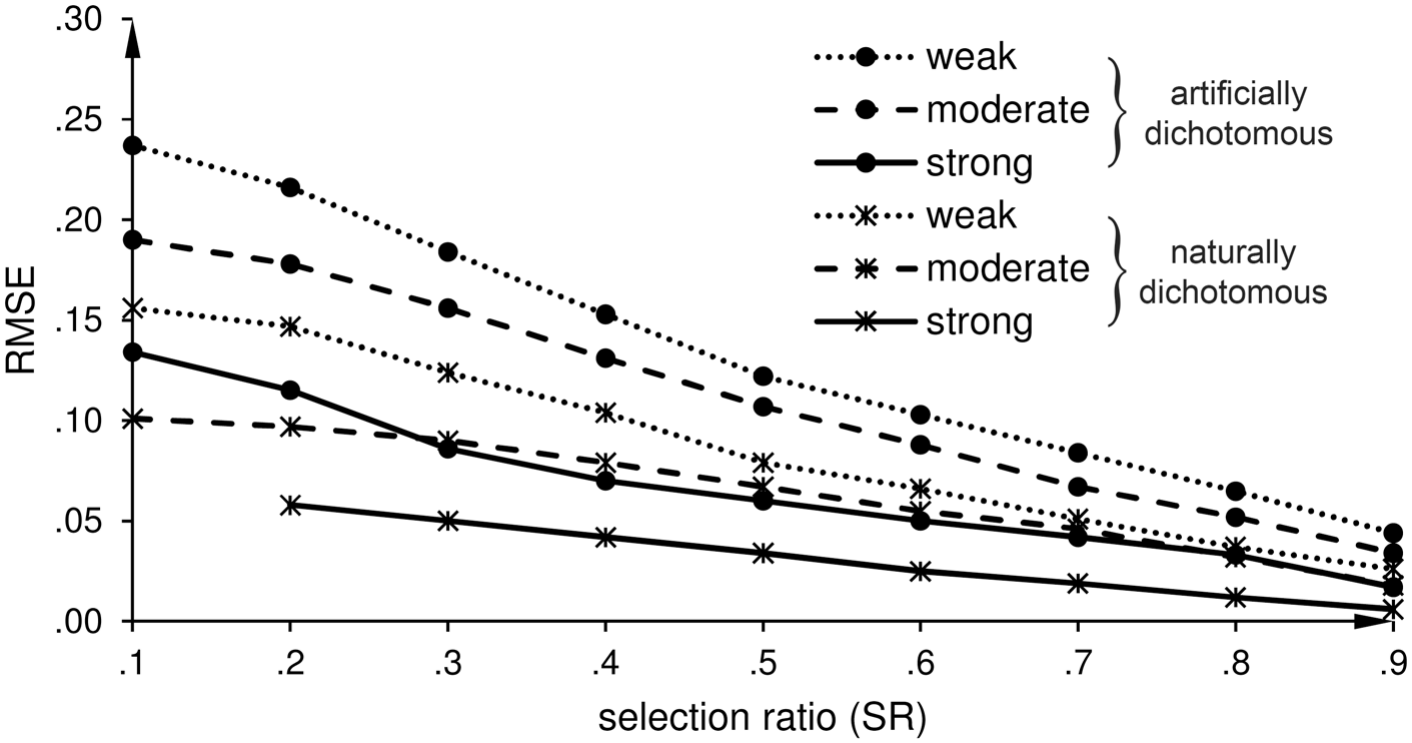
Direct range restriction (DRR): Effects of a weak, moderate, and strong predictive validity on the root mean square error (RMSE) of the estimates of the predictive validity (${\hat{r}}_{\text{b}}$ and ${\hat{r}}_{\text{pb}}$). ${\hat{r}}_{\text{b}}$ is the estimate of the biserial correlation coefficient for an artificially dichotomous criterion variable, and ${\hat{r}}_{\text{pb}}$ is the estimate of the point-biserial correlation coefficient for a naturally dichotomous criterion variable.

**Fig 5:**
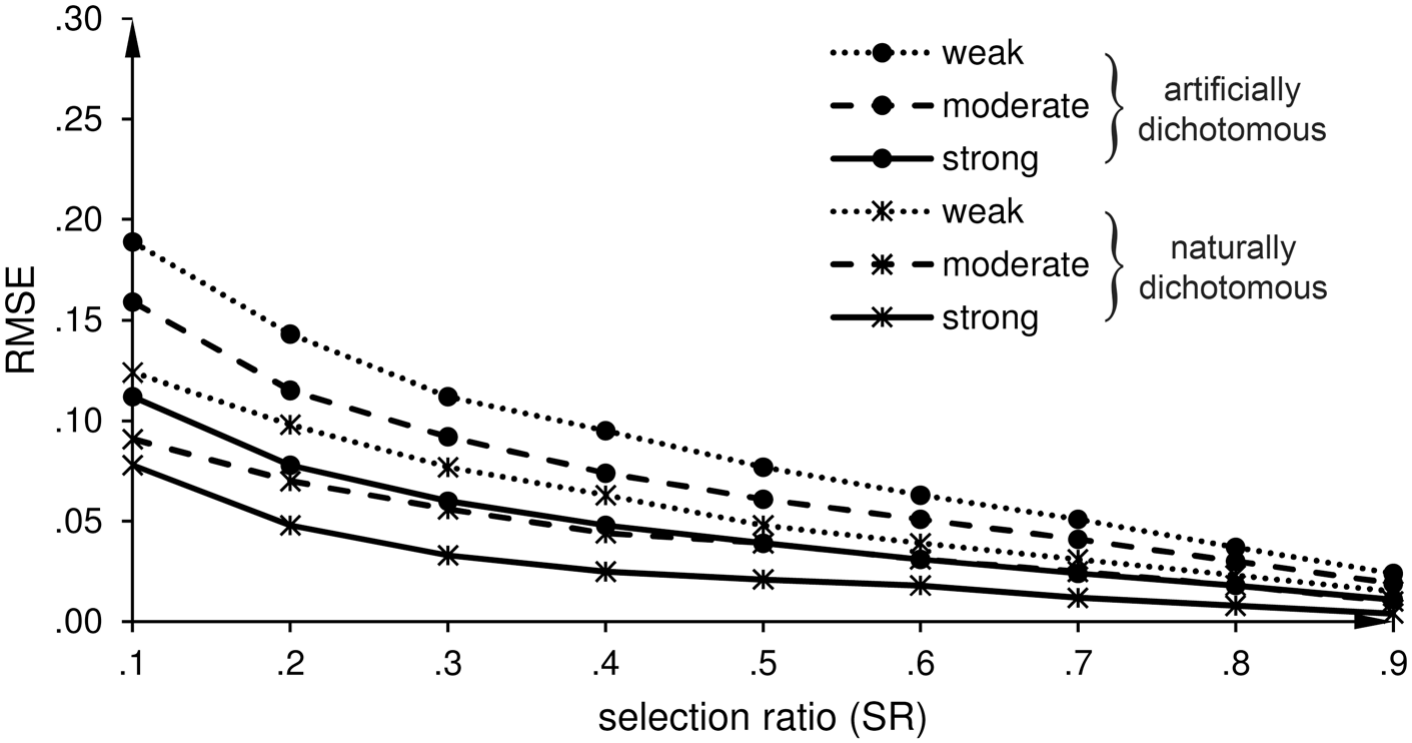
Indirect range restriction (IRR): Effects of a weak, moderate, and strong predictive validity on the root mean square error (RMSE) of the estimates of the predictive validity (${\hat{r}}_{\text{b}}$ and ${\hat{r}}_{\text{pb}}$). **${\hat{r}}_{\text{b}}$** is the estimate of the biserial correlation coefficient for an artificially dichotomous criterion variable, and ${\hat{r}}_{\text{pb}}$ is the estimate of the point-biserial correlation coefficient for a naturally dichotomous criterion variable.

**Fig 6:**
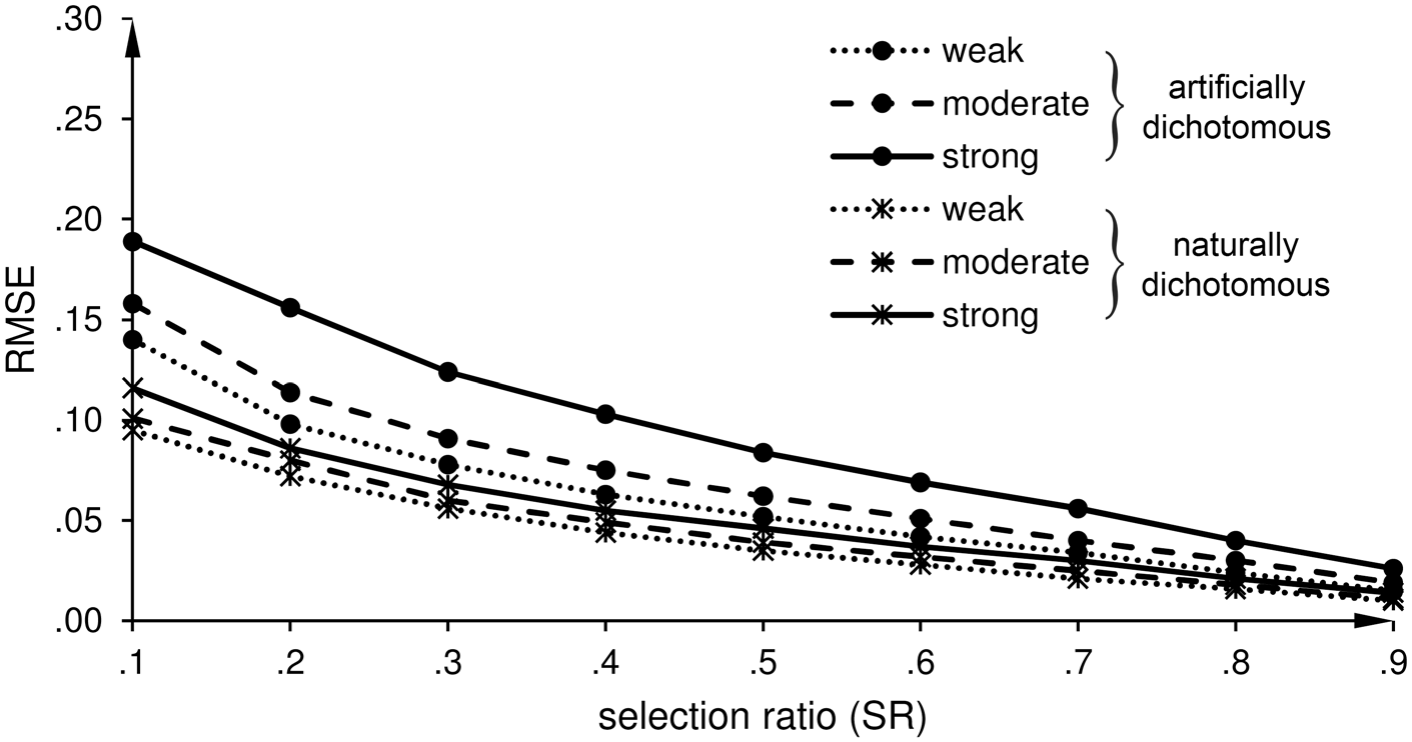
Indirect range restriction (IRR): Effects of a weak, moderate, and strong relationship between predictor *X* and selection variable *Z* on the root mean square error (RMSE) of the estimates of the predictive validity (${\hat{r}}_{\text{b}}$ and ${\hat{r}}_{\text{pb}}$). ${\hat{r}}_{\text{b}}$ is the estimate of the biserial correlation coefficient for an artificially dichotomous criterion variable, and ${\hat{r}}_{\text{pb}}$ is the estimate of the point-biserial correlation coefficient for a naturally dichotomous criterion variable.

Figs 2–6 show the root mean square errors (RMSEs) of the estimated parameters in dependence of the selection ratio (SR). As an overall effect, the accuracy (trueness and precision) of all estimates gradually improved as the SR increased from .1 to .9, i.e. as the loss of criterion data decreased from 90% to 10%. For each purpose, except Purpose 4 that explicitly refers to an IRR scenario, we first display the results for the DRR scenario and then the results for the IRR scenario.

### Purpose 1—Comparison of the two approaches

The first purpose was to compare the correction with multiple imputation by chained equations (MICE) with Thorndike’s correction formulas with regard to the accuracy of the correlation estimates.

*DRR scenario*: Table 3 summarizes the mean errors (MEs) as a measure of the trueness of the predictive validity. The results show that both approaches underestimate the unrestricted correlation at SR of .1 (90% missing values). The underestimation at SR = .1 is larger when correcting with MICE than when correcting with Thorndike’s formula (MICE about -.10; Thorndike about -.05). For SRs beyond .2, the estimates for both kinds of criterion variables are less biased when correcting with MICE. Next, we compared the RMSEs of ${\hat{r}}_{\text{pb}}$ (naturally dichotomous criterion variable) and ${\hat{r}}_{\text{b}}$ (artificially dichotomous criterion variable) when correcting with MICE and with Thorndike’s correction formula for DRR (Eq 1). In the case of ${\hat{r}}_{\text{b}}$, the *F*-ratios range from 1.14 to 2.25 (all *p*s < .001), except at SR = .3 and SR = .4 (*F*-ratios 1.03 and 1.00). In the case of ${\hat{r}}_{\text{pb}}$, the *F*-ratios range from 2.08 to 10.3 (all *p*s < .001), as shown in Table 4. Thus, the correction with MICE is more precise than the correction with Thorndike’s formula (Eq 1) for both kinds of dichotomous criterion variables. The difference in the extent of precision between the two approaches is higher for a naturally dichotomous criterion variable than for an artificially dichotomous criterion variable. Fig 2 shows the RMSEs of both correlation estimates (${\hat{r}}_{\text{b}}\;$ and ${\hat{r}}_{\text{pb}}$) for our proposed correction with multiple imputation by chained equations (MICE) and for the correction with Thorndike’s formula (Eq 1).

**Table 3:** Mean errors (ME) of the correlation estimates.

		Selection ratio (SR)								
		.1	.2	.3	.4	.5	.6	.7	.8	.9
**DRR, artificially dichotomous**	MICE	-.12	-.05	-.02	<.01	<.01	<.01	<.01	<.01	<.01
	Thorndike	-.06	-.05	-.05	-.04	-.04	-.03	-.03	-.02	<.01
**DRR, naturally dichotomous**	MICE	-.09	-.04	-.02	-.01	<.01	<.01	<.01	<.01	<.01
	Thorndike	-.05	-.05	-.05	-.05	-.04	-.03	-.02	-.01	<.01
**IRR, artificially dichotomous**	MICE	-.08	-.03	-.01	<.01	<.01	<.01	<.01	<.01	<.01
	Thorndike	-.04	-.02	-.02	-.01	-.01	-.01	<.01	<.01	<.01
**IRR, naturally dichotomous**	MICE	-.07	-.03	-.02	-.01	<.01	<.01	<.01	<.01	<.01
	Thorndike	-.01	-.03	-.04	-.04	-.03	-.03	-.02	-.01	<.01

${\hat{r}}_{\text{b}}\;$ is the estimate of the biserial correlation coefficient, ${\hat{r}}_{\text{pb}}$ is the estimate point-biserial correlation coefficient.

**Table 4:** *F*-ratio of the correlation estimates when correcting with multiple imputation by chained equations and Thorndike’s formulas.

	Selection ratio (SR)								
	.1	.2	.3	.4	.5	.6	.7	.8	.9
**DRR, artificially dichotomous**	2.23**	1.23**	1.03	1.00	1.14**	1.27**	1.66**	1.95**	2.25**
**DRR, naturally dichotomous**	3.46**	2.08**	2.25**	2.87**	4.24**	5.22**	6.71**	9.19**	10.3**
**IRR, artificially dichotomous**	1.72**	1.46**	1.34**	1.42**	1.48**	1.69**	1.95**	2.40**	2.72**
**IRR, naturally dichotomous**	2.41**	2.68**	3.62**	5.02**	6.76**	8.29**	10.4**	12.4**	12.3**

${\hat{r}}_{\text{b}}$ is the estimate of the biserial correlation coefficient, ${\hat{r}}_{\text{pb}}$ is the estimate point-biserial correlation coefficient, *F*-ratio is calculated by the mean square error (MSE) of the estimate using Thorndike’s formula divided by the MSE of the estimate using MICE, ** *p* < .001.

*IRR scenario*: As shown in Table 3, MICE underestimates the unrestricted correlation for both kinds of criterion variables at SR = .1. However, this bias tends to be smaller for the IRR scenario than for the DRR scenario. With regard to the precision of the estimates, Fig 3 shows that the correlation estimates are more precise for our proposed correction with MICE than for the correction with Thorndike’s formula. The course of the lines is similar to the DRR scenario. The differences in the RMSEs between MICE and Thorndike’s formula are larger for ${\hat{r}}_{\text{pb}}$ than for ${\hat{r}}_{\text{b}}$, as shown in Table 4. The *F*-ratios for ${\hat{r}}_{\text{b}}$ range from 1.34 to 2.72 (all *p*s < .001), and for ${\hat{r}}_{\text{pb}}$ from 2.41 to 12.4 (all *p*s < .001).

### Purpose 2—The effect of the strength of the relationship (X, Y)

The second purpose was to investigate the effect of the strength of the relationship between predictor *X* and criterion *Y* on the accuracy of the correction with MICE. Therefore, we investigated the effect of a weak, moderate, and strong relationship between *X* and *Y* on the precision of the correction with MICE.

*DRR scenario*: Fig 4 shows that the precision of ${\hat{r}}_{\text{pb}}$ and ${\hat{r}}_{\text{b}}$ increases (RMSEs decrease) when the strength of the correlation in the unrestricted dataset increases. In Fig 4, we excluded the value of ${\hat{r}}_{\text{pb}}$ for the condition of SR = .1 combined with a strong relationship between *X* and *Y*, because for this case only three restricted datasets met the prerequisite.

*IRR scenario*: Similar to the DRR scenario, the precision of the estimated correlation coefficient for naturally and artificially dichotomous criterion variables increases when the strength of the relationship between *X* and *Y* increases, as shown in Fig 5.

### Purpose 3—Correcting the biased base rate of success (BR)

The third purpose was to investigate the accuracy of the correction of the biased BR with MICE. Table 5 summarizes the MEs and the RMSEs of the estimate of the base rate of success ($\hat{BR}$) for the two scenarios and both kinds of criterion variables.

**Table 5:** Accuracy of the estimate of the base rate of success when correcting via multiple imputation by chained equations (MICE).

		Selection ratio (SR)								
		.1	.2	.3	.4	.5	.6	.7	.8	.9
**DRR, artificially dichotomous**	ME	.07	.01	<.01	<.01	<.01	<.01	<.01	<.01	<.01
	RMSE	.157	.120	.090	.066	.047	.034	.022	.014	.007
**DRR, naturally dichotomous**	ME	.07	.02	<.01	<.01	<.01	<.01	<.01	<.01	<.01
	RMSE	.154	.111	.081	.059	.040	.028	.018	.011	.005
**IRR, artificially dichotomous**	ME	.07	.01	<.01	<.01	<.01	<.01	<.01	<.01	<.01
	RMSE	.151	.113	.084	.061	.045	.031	.020	.012	.006
**IRR, naturally dichotomous**	ME	.08	.02	<.01	<.01	<.01	<.01	<.01	<.01	<.01
	RMSE	.142	.108	.078	.054	.037	.026	.016	.010	.005

DRR is the direct range restriction, IRR is the indirect range restriction, ME is the mean error, and RMSE is the root mean square error.

*DRR scenario*: The mean errors in Table 5 show an overestimation of the base rate of success (+.07) at an SR of .1 for both kinds of criterion variables. This effect is contrary to the correlation estimates, which underestimate the unrestricted correlation. For SRs beyond .2, the estimates are not biased. In the same manner as for the estimation of the correlation coefficients, the RMSEs of $\hat{BR}$ decreases as the selection ratio increases (from .157 to .007 for an artificially dichotomous criterion variable and from .154 to .005 for a naturally dichotomous one).

*IRR scenario*: The results for the IRR scenario are similar to the DRR scenario. The MEs show an overestimation of the base rate of success only at an SR of .1 and the precision of $\hat{BR}$ increases as the selection ratio decreases.

### Purpose 4—The effect of the strength of the relationship (*Z*, *X*)

The fourth purpose of this study was to investigate the effect of the strength of the relationship between *Z* and *X* on the accuracy of the correction with MICE in an IRR scenario. Therefore, we investigated the effect of a weak, moderate, and strong relationship between the selection variable *Z* and the predictor variable *X* on the precision of the correction with MICE. The results in Fig 6 show that the precision of the estimates increases (RMSEs decrease) when the relationship between *Z* and *X* decreases.

## Discussion

A recurring methodological problem in the evaluation of the predictive validity of selection methods is the loss of data for the criterion variable. This so-called range restriction problem results in biased population estimates because the observed sample (the selected sample) is not representative of the population of interest (the applicant population). Hence, these biased estimates have to be corrected. However, researchers have almost exclusively focused on correction in the case of a continuous criterion variable. Therefore, our aim was to propose an approach for correcting for range restriction when the criterion variable is dichotomous. We applied this approach to the two most common selection scenarios in personnel selection and higher education: a direct range restriction scenario (DRR) and an indirect range restriction scenario (IRR). We investigated two kinds of dichotomous criterion variables: artificially and naturally dichotomous criterion variables.

The proposed approach correcting for range restriction is to view the selection as a missing data mechanism. We used multiple imputation by chained equations (MICE), which is a state-of-the-art method for dealing with missing data. We pointed out the importance of the unknown base rate of success, which has to be considered when correcting for range restriction in the case of a dichotomous criterion. The proposed approach corrects for range restriction by replacing the missing values of the criterion variable before estimating the predictive validity and the BR at the same time.

We investigated the accuracy of the proposed correction by conducting Monte Carlo simulations, which allowed us to compare the parameter estimates with the true parameters in an experimental design. In the present simulation study, we varied several factors (correlations, base rate of success, and selection ratio) over a wide range in order to examine the accuracy of the correction for a variety of possible datasets.

We compared our proposed missing data approach with Thorndike's formulas (established for a continuous criterion) in terms of the accuracy of the parameter estimates. The Monte Carlo simulations show that our proposed approach performs effectively in both the DRR scenario and the IRR scenario. The correction of the biased predictive validity with MICE is more precise than the correction with Thorndike’s formulas. Furthermore, we were able to show that the missing data approach provides a valid estimate of the base rate of success that has not been considered in the scientific literature. On the basis of our findings, we argue for the use of multiple imputation by chained equations in the evaluation of the predictive validity of selection methods when the criterion is dichotomous. To our knowledge, the proposed correction for range restriction using multiple imputation by chained equations is the first approach that provides a proper correction for the biased predictive validity when the criterion variable is dichotomous. The missing data approach facilitates the correction of the biased correlation coefficient as well as of the unknown base rate of success.

Some limitations of our study should be mentioned that open the field for further research. In the simulation study, we used a small data matrix of two variables in the DRR scenario and of three variables in the IRR scenario [7]. As in simulation studies, it is often difficult to generate multivariate random correlated datasets, especially for multivariate non-normal distributions. Further research should examine the effect of a data matrix with more variables on the accuracy of the correction. In the present study, we investigated the accuracy of the correction for one sample size. However, one important research question with regard to the multiple imputation by chained equations approach is how small the total sample size as well as the restricted sample size can be for a precise and unbiased correction. We recommend investigating these limitations in further studies.

The IRR scenario assumes that the selection variable *Z* is measured. In this case, the missing data mechanism is MAR (ignorable selection process, [3]), and therefore we can use a multiple imputation technique. However, in cases of incidental selections, *Z* is sometimes either partially measured or unmeasured. For example, this is the case when selection is based on an unquantified subjective judgment, or in the case of self-selection, when individuals remove themselves from a sample for reasons that are not measured. In such cases, the missing data mechanism is missing not at random (MNAR, non-ignorable selection process). In statistics, this methodological problem is known as sample selection bias [3]. Traditional range restriction corrections yield unsatisfactory estimates of *r*_*XY*_ when the selection process is non-ignorable [3,39]. A correction procedure for selection bias for a continuous dependent variable has been developed in the field of economics [57,58]. Muthén and Hsu [59] presented a latent variable model. In cases of non-ignorable selection, further studies should examine the accuracy of this latent variable model for a dichotomous criterion using weighted least squares means and variance adjusted (WLSMV). As another approach, MICE can also be used to correct for MNAR data [55].

Some recommendations for practitioners and organizations can be derived from our research. Sometimes, test data from applicants who were not selected are discarded are not available in later validity studies. However, discarding applicants’ test data leads to a needless loss of information regarding the predictive validity of selection methods. Therefore, we recommend that organizations store the data in an anonymized form for future evaluations of the predictive validity. Although our approach is applicable for data with up to 90% missing values, we urge caution in the interpretation of the estimates when missing values exceed 70%.

In summary, the results from the simulation study show that the proposed correction with multiple imputation by chained equations is effective in correcting for DRR and IRR scenarios when the criterion variable is dichotomous. Therefore, the approach presented in this paper seems to be promising in terms of overcoming recurring range restriction problems in the evaluation of the predictive validity of selection methods.

## Supporting Information

S1 Data Direct range restriction—artificially dichotomous criterion variable. (ZIP)

S2 Data Direct range restriction—naturally dichotomous criterion variable. (ZIP)

S3 Data Indirect range restriction—artificially dichotomous criterion variable. (ZIP)

S4 Data Indirect range restriction—naturally dichotomous criterion variable. (ZIP)

S1 Rscript Direct range restriction—artificially dichotomous criterion variable. (DOCX)

S2 Rscript Direct range restriction—naturally dichotomous criterion variable. (DOCX)

S3 Rscript Indirect range restriction—artificially dichotomous criterion variable. (DOCX)

S4 Rscript Indirect range restriction—naturally dichotomous criterion variable. (DOCX)
