# Supplementary material for: A Missing Data Approach to Correct for Direct and Indirect Range Restrictions with a Dichotomous Criterion: A Simulation Study
Source: PLoS One. 2016 Mar 28;11(3):e0152330. doi: 10.1371/journal.pone.0152330 (PMC4809486; doi:10.1371/journal.pone.0152330)
Supplement: S1 Rscript — (DOCX) [file pone.0152330.s005.docx]

#########################################################

library(ltm)

library(mice)

library(psych)

N = 500 # Sample Size

EXPERIMENTS = 5000 # number of experiments

selratio <- c(.1,.2,.3,.4,.5,.6,.7,.8,.9) # Selection ratio

resultsL = EXPERIMENTS*length(selratio)

print(resultsL)

results <- matrix(nrow=resultsL, ncol=25)

colnames(results) <- c("experiment","N","selratio","pearsonURxy","rbURxy","n1UR","n0UR","qUR","sdxUR","n1RR","qRR","sdxRR","rbRR","Tpq","qRRres","qMice","VBqMice","b0Mice","b1Mice","rbAprxT","rbMice","VBrbMice","qResMice","rbResT","rbResMice")

#########################################################

## BEGIN SIMULATION

index = 0

for(iExperiment in 1:EXPERIMENTS){ # start Experiments-loop

print(iExperiment)

flush.console()

## GENERATE DATA

Sigma = matrix(c(1,2,3,4), 2, 2)

while(det(Sigma) < 0){

rxy = runif(1, .1, .9) # initial random Pearson Correlation(X, Y)

Sigma = matrix(c(1, rxy, rxy, 1), 2, 2)

}

data <- data.frame(mvrnorm(N, c(0, 0), tol=1e-05, Sigma, empirical=TRUE))

colnames(data) <- c("x", "y")

pearsonURxy = cor(data$x, data$y)

## DICHOTOMISE

q = runif(1, .1, .9) # intitial random value of the base rate of success

cutoff = qnorm(q) # cutoff for dichotomisation

data$y <- ifelse(data$y < cutoff, FALSE, TRUE)

n1UR = sum(data$y) # number of successful cases (unrestricted)

n0UR = N - n1UR

qUR = mean(data$y) # base rate of success (unrestricted)

rbURxy = biserial(data$x, data$y)

## SORT in DESCENDING ORDER to X

data <- data[with(data, order(-data$x)),]

### SELECTION - DIRECT RANGE RESTRICTION

for(isr in 1:length(selratio) ){ # start SR-loop

index = index + 1

## GENERATE SYSTEMATIC MISSINGS

dataRR <- data

dataRR$y[((selratio[isr]*N)+1):N] = NA

n1RR = sum(na.omit(dataRR$y[1:N])) # number of successful cases (restricted)

n0RR = selratio[isr]*N - n1RR

qRR = mean(na.omit(dataRR$y[1:N])) # success rate (restricted)

results[index, "sdxUR"] = sd(data$x)

results[index, "sdxRR"] = sd(dataRR[1:(selratio[isr]*N), "x"])

rbRR = biserial(dataRR$x[1:(selratio[isr]*N)], dataRR$y[1:(selratio[isr]*N)])

results[index, "Tpq"] = (selratio[isr]*N)*qRR*(1-qRR)

## ESTIMATION

if(n1RR > 5 && n0RR > 5){

noImp = 20 # number of imputations

## MICE

miceFit <- mice(dataRR, meth=c("","logreg"), m=noImp)

miceFit.out <- with(data=miceFit, exp=glm(y~x, family=binomial(link="probit")))

## BASE RATE of SUCCESS and BISERIAL CORRELATION COEFFICIENT (MICE)

qkMice = NA; rbkMice = NA

for(kk in 1:noImp){

qkMice[kk] = mean(complete(miceFit, kk)$y)

rbkMice[kk] <- biserial(complete(miceFit,kk)$x, complete(miceFit,kk)$y)

}

results[index, "qMice"] = mean(qkMice)

results[index, "VBqMice"] = var(qkMice) # Variance between imputations

results[index, "qResMice"] = results[index, "qMice"] - qUR

results[index, "rbMice"] = mean(rbkMice)

results[index, "VBrbMice"] = var(rbkMice) # Variance between imputations

results[index, "rbResMice"] = results[index, "rbMice"] - rbURxy

## THORNDIKE's CORRECTION FORMULA (rb)

sdxUR = results[index, "sdxUR"]

sdxRR = results[index, "sdxRR"]

results[index,"rbAprxT"] = (sdxUR*rbRR)/sqrt(sdxUR^2*rbRR^2+sdxRR^2-sdxRR^2*rbRR^2)

results[index,"rbResT"] = results[index,"rbAprxT"] - rbURxy

}

else{

results[index, "qMice"] = NA

results[index, "rbAprxT"] = NA

results[index, "rbMice"] = NA

results[index, "VBqMice"] = NA

results[index, "VBrbMice"] = NA

results[index, "qResMice"] = NA

results[index, "rbResT"] = NA

results[index, "rbResMice"] = NA

}

## CALCULATE RESULTS

results[index, "experiment"] = iExperiment

results[index, "N"] = N

results[index, "selratio"] = selratio[isr]

results[index, "pearsonURxy"] = pearsonURxy

results[index, "rbURxy"] = rbURxy

results[index, "n1UR"] = n1UR

results[index, "n0UR"] = n0UR

results[index, "qUR"] = qUR

results[index, "n1RR"] = n1RR

results[index, "qRR"] = qRR

results[index, "qRRres"] = qRR - qUR

results[index, "rbRR"] = rbRR

results[index, "b0Mice"] = pool(miceFit.out)[8]$qbar[1]

results[index, "b1Mice"] = pool(miceFit.out)[8]$qbar[2]

} # end SR-loop

} # end Experiments-loop

## MAKE OUTPUT-FILE

write.table(results, file = "C:/…”, # Please, enter path and file name (.txt)

append = FALSE, quote = TRUE, sep = ";",

eol = "\n", na = "", dec = ".", row.names = TRUE,

col.names = TRUE, qmethod = c("escape", "double"),

fileEncoding = "")

print("PROGRAM END!!!")
