# Supplementary material for: A Missing Data Approach to Correct for Direct and Indirect Range Restrictions with a Dichotomous Criterion: A Simulation Study
Source: PLoS One. 2016 Mar 28;11(3):e0152330. doi: 10.1371/journal.pone.0152330 (PMC4809486; doi:10.1371/journal.pone.0152330)
Supplement: S4 Rscript — (DOCX) [file pone.0152330.s008.docx]

#########################################################

library(ltm)

library(mice)

library(psych)

##

N = 500 # Sample size

EXPERIMENTS = 5000 # Number of experiments

selratio <- c(.1,.2,.3,.4,.5,.6,.7,.8,.9) # Selection ratio

resultsL = EXPERIMENTS*length(selratio)

print(resultsL)

results <- matrix(nrow=resultsL, ncol=26)

colnames(results) <- c("experiment","N","selratio","pearsonURzx","rpbURxy","rpbURzy","n1UR","n0UR","qUR","n1RR","qRR","rpbRRxy","rpbRRzy","pearsonRRzx","Tpq","qRRres","qMice","VBqMice","b0Mice","b1Mice","rpbAprxT","rpbMice","VBrpbMice","qResMice","rpbResT","rpbResMice")

#########################################################

## BEGIN SIMULATION

index = 0

for(iExperiment in 1:EXPERIMENTS){ # start Experiments-loop

print(iExperiment)

flush.console()

## GENERATE DATA

# Initialise base rate of success

q = runif(1, .1, .9)

n1UR = round(N*q, 0)

n0UR = N - n1UR

# Initialise correlations

pearsonzx = runif(1, .1, .9) # initial random Pearson correlation (Z, X)

rpbxy = runif(1, .1, .9) # initial random point-biserial correlation (X, Y)

rpbzy = runif(1, .1, .9) # initial random point-biserial correlation (Z, Y)

Sigma = matrix(c(1, pearsonzx, pearsonzx, 1), 2 , 2)

dxy = rpbxy / sqrt(n0UR*n1UR/N^2)

dzy = rpbzy / sqrt(n0UR*n1UR/N^2)

y <- NULL

y[1:n0UR] <- FALSE; y[(n0UR+1):N] <- TRUE

data0 <- data.frame(mvrnorm(n0UR, c(0, 0), tol=1e-03, Sigma, empirical=TRUE))

rpbURxy = 0; rpbURzy = 0; pearsonURzx = 0;

while((abs(rpbURxy-rpbxy) > .01) && (abs(rpbURzy-rpbzy) > .01) && (abs(pearsonURzx-pearsonzx) > .01)){

data1 <- data.frame(mvrnorm(n1UR, c(dxy, dzy), tol=1e-03, Sigma))

data <- data.frame(y, rbind(data0, data1))

colnames(data) <- c("y", "x", "z")

dxy = rpbxy / sqrt(n0UR*n1UR/N^2) * sd(data$x)

dzy = rpbzy / sqrt(n0UR*n1UR/N^2) * sd(data$z)

rpbURxy = biserial.cor(data$x, data$y, level = 2)

rpbURzy = biserial.cor(data$z, data$y, level = 2)

pearsonURzx = cor(data$z, data$x)

}

data <- data.frame("y"=data$y, "x"=scale(data$x, TRUE, TRUE), "z"=scale(data$z, TRUE, TRUE))

qUR = n1UR/N # base rate of success (unrestricted)

rpbURxy = biserial.cor(data$x, data$y, level = 2)

rpbURzy = biserial.cor(data$z, data$y, level = 2)

pearsonURzx = cor(data$z, data$x)

## SORT in DESCENDING ORDER to Z

data <- data[with(data, order(-data$z)),]

### SELECTION – INDIRECT RANGE RESTRICTION

for(isr in 1:length(selratio) ){ # start SR-loop

index = index + 1

## GENERATE SYSTEMATIC MISSINGS

dataRR <- data.frame("x"=data$x, "y"=data$y, "z"=data$z)

dataRR$y[((selratio[isr]*N)+1):N] = NA

n1RR = sum(na.omit(dataRR$y[1:N])) # number of successful cases (restricted)

n0RR = selratio[isr]*N - n1RR

qRR = mean(na.omit(dataRR$y[1:N])) # success rate (restricted)

sd_zUR = sd(data$z)

sd_zRR = sd(dataRR[1:(selratio[isr]*N), "z"])

rpbRRxy = biserial.cor(dataRR$x[1:(selratio[isr]*N)], dataRR$y[1:(selratio[isr]*N)], level = 2)

rpbRRzy = biserial.cor(dataRR$z[1:(selratio[isr]*N)], dataRR$y[1:(selratio[isr]*N)], level = 2)

pearsonRRzx = cor(dataRR$z[1:(selratio[isr]*N)], dataRR$x[1:(selratio[isr]*N)])

results[index, "Tpq"] = (selratio[isr]*N)*qRR*(1-qRR)

## ESTIMATION

if(n1RR > 5 && n0RR > 5){

noImp = 20 # number of imputations

## MICE

miceFit <- mice(dataRR, meth=c("","logreg",""), m=noImp)

miceFit.out <- with(data=miceFit, exp=glm(y~x+z, family=binomial(link="logit")))

## BASE RATE of SUCCESS and POINT-BISERIAL CORRELATION COEFFICIENT (MICE)

qkMice = NA; rpbkMice = NA

for(kk in 1:noImp){

qkMice[kk] = mean(complete(miceFit, kk)$y)

rpbkMice[kk] <- biserial.cor(complete(miceFit,kk)$x, complete(miceFit,kk)$y, level = 2)

}

results[index, "qMice"] = mean(qkMice)

results[index, "VBqMice"] = var(qkMice) # Variance between imputations

results[index, "qResMice"] = results[index, "qMice"] - qUR

results[index, "rpbMice"] = mean(rpbkMice)

results[index, "VBrpbMice"] = var(rpbkMice) # Variance between imputations

results[index, "rpbResMice"] = results[index, "rpbMice"] - rpbURxy

## THORNDIKE's CORRECTION FORMULA (rpb)

numerator = rpbRRxy+pearsonRRzx*rpbRRzy*(sd_zUR^2/sd_zRR^2-1)

denominator = sqrt(1+pearsonRRzx^2*(sd_zUR^2/sd_zRR^2-1))*sqrt(1+rpbRRzy^2*(sd_zUR^2/sd_zRR^2-1))

results[index,"rpbAprxT"] = numerator / denominator

results[index,"rpbResT"] = results[index,"rpbAprxT"] - rpbURxy

}

else{

results[index, "qMice"] = NA

results[index, "rpbAprxT"] = NA

results[index, "rpbMice"] = NA

results[index, "VBqMice"] = NA

results[index, "VBrpbMice"] = NA

results[index, "qResMice"] = NA

results[index, "rpbResT"] = NA

results[index, "rpbResMice"] = NA

}

## CALCULATE RESULTS

results[index, "experiment"] = iExperiment

results[index, "N"] = N

results[index, "selratio"] = selratio[isr]

results[index, "rpbURxy"] = rpbURxy

results[index, "rpbURzy"] = rpbURzy

results[index, "pearsonURzx"] = pearsonURzx

results[index, "n1UR"] = n1UR

results[index, "n0UR"] = n0UR

results[index, "qUR"] = qUR

results[index, "n1RR"] = n1RR

results[index, "qRR"] = qRR

results[index, "qRRres"] = qRR - qUR

results[index, "rpbRRxy"] = rpbRRxy

results[index, "rpbRRzy"] = rpbRRzy

results[index, "pearsonRRzx"] = pearsonRRzx

results[index, "b0Mice"] = pool(miceFit.out)[8]$qbar[1]

results[index, "b1Mice"] = pool(miceFit.out)[8]$qbar[2]

} # end SR-loop

} # end Experiments-loop

## MAKE OUTPUT-FILE

write.table(results, file = "C:/…", # Please, enter path and file name (.txt)

append = FALSE, quote = TRUE, sep = ";",

eol = "\n", na = "", dec = ".", row.names = TRUE,

col.names = TRUE, qmethod = c("escape", "double"),

fileEncoding = "")

print("PROGRAM END!!!")
